# Opening the black box of traumatic brain injury: a holistic approach combining human 3D neural tissue and an *in vitro* traumatic brain injury induction device

**DOI:** 10.3389/fnins.2023.1189615

**Published:** 2023-06-15

**Authors:** Céline Loussert-Fonta, Luc Stoppini, Yoan Neuenschwander, Ophélie Righini, Denis Prim, Cédric Schmidt, Marc O. Heuschkel, Loris Gomez Baisac, Milica Jovic´, Marc E. Pfeifer, Jérôme Extermann, Adrien Roux

**Affiliations:** ^1^Tissue Engineering Laboratory, HEPIA HES-SO University of Applied Sciences and Arts Western Switzerland, Geneva, Switzerland; ^2^Micro-Nanotechnology Group, HEPIA HES-SO University of Applied Sciences and Arts Western Switzerland, Geneva, Switzerland; ^3^Diagnostic Systems Research Group, Institute of Life Technologies, School of Engineering, University of Applied Sciences and Arts Western Switzerland (HES-SO Valais-Wallis), Sion, Switzerland

**Keywords:** traumatic brain injury, optical projection tomography, 3D neural tissue, multiplex protein assays, biomarkers, electrophysiology, *in vitro* model

## Abstract

Traumatic brain injury (TBI) is caused by a wide range of physical events and can induce an even larger spectrum of short- to long-term pathophysiologies. Neuroscientists have relied on animal models to understand the relationship between mechanical damages and functional alterations of neural cells. These *in vivo* and animal-based *in vitro* models represent important approaches to mimic traumas on whole brains or organized brain structures but are not fully representative of pathologies occurring after traumas on human brain parenchyma. To overcome these limitations and to establish a more accurate and comprehensive model of human TBI, we engineered an *in vitro* platform to induce injuries via the controlled projection of a small drop of liquid onto a 3D neural tissue engineered from human iPS cells. With this platform, biological mechanisms involved in neural cellular injury are recorded through electrophysiology measurements, quantification of biomarkers released, and two imaging methods [confocal laser scanning microscope (CLSM) and optical projection tomography (OPT)]. The results showed drastic changes in tissue electrophysiological activities and significant releases of glial and neuronal biomarkers. Tissue imaging allowed us to reconstruct the injured area spatially in 3D after staining it with specific nuclear dyes and to determine TBI resulting in cell death. In future experiments, we seek to monitor the effects of TBI-induced injuries over a prolonged time and at a higher temporal resolution to better understand the subtleties of the biomarker release kinetics and the cell recovery phases.

## Introduction

Traumatic brain injury (TBI) is the leading cause of mortality in young adults and a significant cause of death and disability across all countries, especially in low- and middle-income countries (Maas et al., 2017; Dewan et al., 2019; Schweitzer et al., 2019). TBI occurs when an external mechanical force is applied to the brain, possibly leading to permanent or temporary impairment of cognitive and physical functions. The severity of the trauma has been classified using the Glasgow Coma Scale (GCS). GCS scores of 13–15 represent mild brain injuries, 9–12 are moderate, and 3–8 are severe (Schweitzer et al., 2019). A recent report estimated the global incidence of all-severity and all-cause TBI at 939 cases per 100,000 people, representing 69 million worldwide. Mild TBI (mTBI) represents 740 cases per 100,000 people, representing 55.9 million people annually (Dewan et al., 2019). The spectrum of symptoms experienced by mTBI patients is vast, ranging from a slight headache to loss of consciousness (<30 min) and even post-traumatic amnesia (<24 h) (Blyth and Bazarian, 2010). At the brain tissue level, the impact leads to short-term primary injuries characterized by structural damages to blood–brain barrier (Cash and Theus, 2020), axonal injury (Tang-Schomer et al., 2012; Johnson et al., 2013), and microglial activation and microhemorrhages (Oppenheimer, 1968). Secondary injuries might develop over minutes to months after the primary lesions and are catalyzed by excessive excitatory neurotransmitter release and calcium influx, leading to apoptotic cell death or/and even early onset of neurodegenerative diseases, which can induce, in the long term, neurodegenerative diseases (Bramlett and Dietrich, 2015; Ng and Lee, 2019; Jarrahi et al., 2020; Dodd et al., 2022).

Despite the increasing awareness about the possible long-term damaging effects of mTBI, improvements in diagnosis and treatment are still insufficient. This concern is mainly due to the limited understanding of the primary and secondary injury mechanisms contributing to long-term sequels (Liaudanskaya et al., 2020). Therefore, to better understand the physiopathology of this type of trauma, various models have been developed, which mainly involve rodents or rodent tissues and can be categorized based on the type of injury sustained (focal or diffuse lesion) or the technique used (such as closed-skull weight drop or lateral fluid impact injury) (Kabadi et al., 2010; Arun et al., 2011; Morrison et al., 2011; Osier and Dixon, 2016). However, these models' lack of control over internal tissue and cell-level biomechanics may exacerbate animal-to-animal variability and do not fully illustrate human physiopathology (Azkona and Sanchez-Pernaut, 2022). Recent technological advancements have allowed the creation of 3D brainlike structures called cerebral organoids (Chiaradia and Lancaster, 2020), which resemble the cellular and anatomical composition of different regions of the human brain and can mimic disease pathways (Lancaster et al., 2013; Mariani et al., 2015; Garcez et al., 2016; Pollen et al., 2019; Velasco et al., 2019). Human brain organoids are becoming an emerging tool in TBI and mTBI as they provide a window into the injured tissues after trauma (Jgamadze et al., 2020; Ramirez et al., 2021a,b) and over time into the chronic phase of injury (Silvosa et al., 2022).

In this study, we developed an *in vitro* platform, termed “*in vitro* TBI,” inducing injury by a finely tunable (velocity and force) expulsion of liquid using a microvalve onto bioengineered human 3D neural tissues. In addition, we propose a correlative workflow to characterize in detail the traumatic area by combining electrical monitoring, imaging [confocal microscopy and optical projection tomography (OPT)], and circulating biomarkers analysis by multiplex assays (Jović et al., 2022).

With this approach, we could evaluate the significant changes occurring after the impact, such as electrophysiological dysregulation, tissue damage and loss, and temporal releases of multiple biomarkers. As a perspective, we hope to monitor TBI and mTBI with a higher temporal resolution to understand better the exact biomarker release kinetics and the tissue recovery processes. The ultimate objective was to design a rapid and sensitive diagnostic tool for mTBI and support the development of therapeutic and potentially neuroprotective agents while identifying appropriate dosing and toxicity levels.

## Materials and methods

### 3D neural tissue generation

Neural stem cells derived from induced pluripotent stem cells (NSChIPSC) (#A3890101 Thermo Fisher) were seeded with a density of 25,000 cells/cm^2^ in six-well plates and processed for the generation of 3D neural tissue according to the protocol previously described in Govindan et al. (2021). For all the experiments, 1-year-old 3D neural tissues were used.

### TBI-induction platform: description and parameters

**Figure 1:**
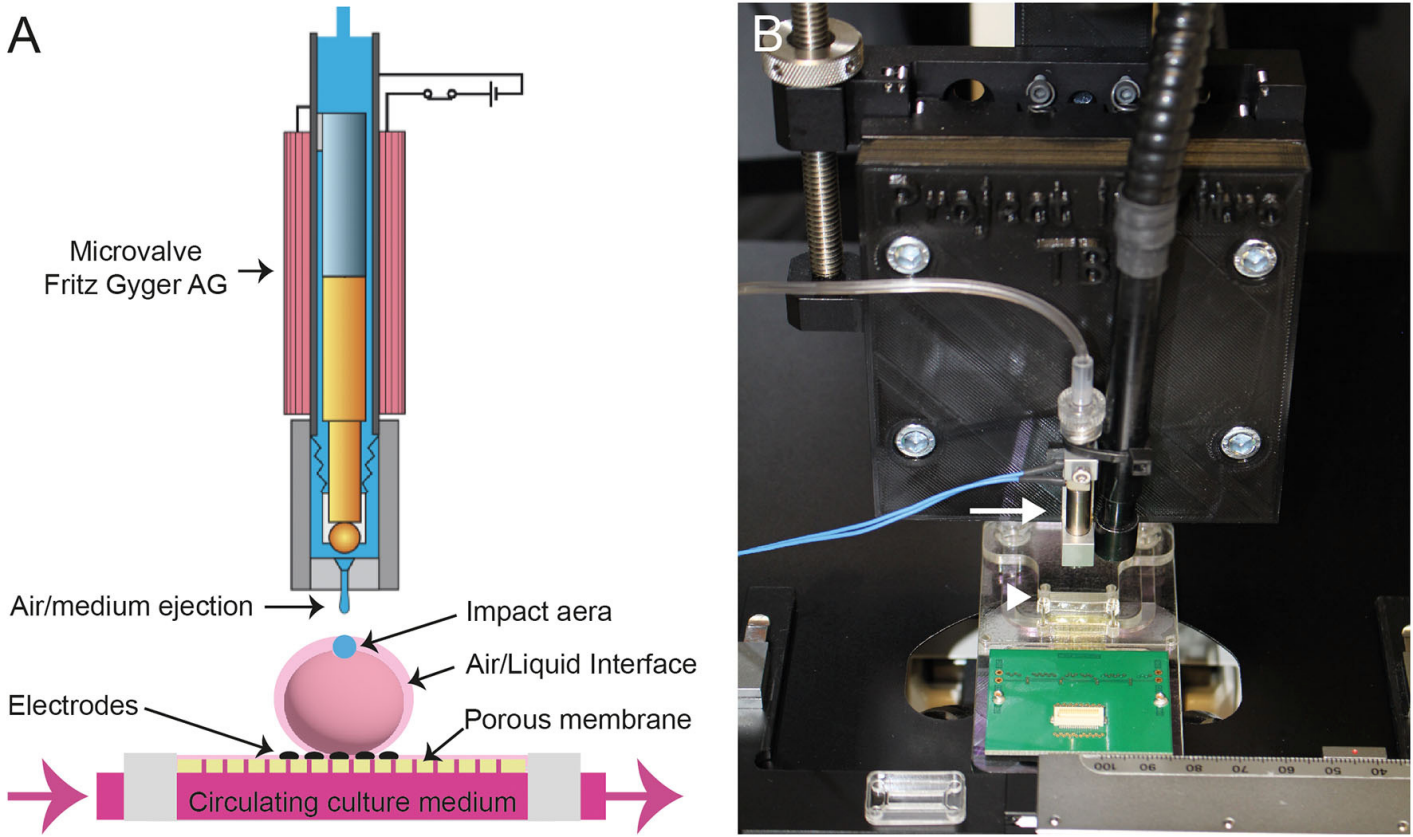
**(A)** Scheme of the Fritz Gyger microvalve (modified with the accord of Fritz Gyger AG) ejecting a drop of culture medium on a 3D neural tissue placed onto a porous Multi-Electrode Array (MEA) biochip. **(B)** Picture showing the microvalve (white arrow) attached to an XYZ translation stage mounted on an inverted microscope. The MEA is indicated with a white arrowhead.

The developed instrument platform is based on a microvalve (#SMLD 300G, Fritz Gyger AG) that creates impacts on the neural tissue with either sterile air or culture medium. The microvalve is supplied with a continuous flow of compressed air (5 bars). The microvalve is mounted on a manual translation stage (#LT1, Thorlabs Inc.), which was fixed on an inverted microscope (#Axiovert 25, Zeiss). The position of the targeted 3D neural tissue, positioned on a Multi-Electrode Array (MEA) biochip or 24- or 96-well microplates, is reached due to an XY translation stage that is attached to the microscope's plate. This overall system allows to perform precise injuries on the targeted sample (Figure 1 and Supplementary Figure 1). In addition, we have developed a dedicated protocol to sterilize the entire system of the ejection of culture medium allowing the follow-up of the traumatized neural tissues during several days.

The microvalve is controlled by a programmable logic controller (#VC Mini, Fritz Gyger AG), and software (#MVC, Fritz Gyger AG) that allows the setting of the different parameters: Open time = the amount of time the microvalve is open; Cycle time = the total time required for one shot (ejection of culture medium or air); number of cycles = number of shots; the peak current = current that goes through the coil; and finally, the peak time = time for which the valve is driven with an increased current for an instantaneous opening and corresponds to the time taken for the valve to open fully. For the experiments described in this work, we have used the following parameters: the open time was set at 1 ms and the cycle time at 100 ms with single shots.

### Electrophysiology data acquisition system and brain tissue interface

3D neural tissues used in this work were also monitored electrically to track the functional effect of mTBI. To interface the 3D neural tissue with recording electrodes, proprietary 32-channel MEA biochips and data acquisition systems have been used. MEA biochips were specifically adapted to allow air–liquid interface culture of the 3D neural tissue, providing optimal conditions for long-term tissue survival. MEA biochips were made of porous membranes incorporating thin film recording microelectrodes on which the 3D neural tissues are placed onto. A fluidic chamber below the membrane allows to feed the tissue with a culture medium sucked by capillary forces, thus allowing the tissue to remain supplied and humidified while staying at the air–liquid interface on the top of the membrane.

The MEA biochips were made of four microfabricated polyimide membrane strips (thickness of 8 μm and an equivalent porosity of 10% of the working area achieved by holes of ø 7.5 μm on a 20-μm grid) that incorporate eight low-impedance platinum black electrodes (ø 30 μm, located on a 200-μm grid, impedance below 100 kΩ at 1 kHz) in each. These strips were mounted using conductive glue onto a printed circuit board that allows connection to external signal amplification and data acquisition electronics. The fluidic chamber below the membranes was made of several layers of laser-cut poly(methyl)methacrylate parts assembled using adhesive transfer tape (#*467MP*, 3M). The MEA biochips have been described previously (Ferlauto et al., 2018; Wertenbroek et al., 2021).

The functional neural electrical activity was amplified using two 16-channel Digital Electrophysiology Stimulator/Amplifier Chips (#RHS2116, Intan technologies), and data were acquired, displayed, and saved using the proprietary “Spike-on-Chip” platform (Wertenbroek et al., 2021). Data pre-analysis was performed using the Spike-on-Chip software, and home-made software was used to generate spike raster plots and signal figures from the acquired data.

### Electrophysiology experimental protocol

**Figure 2:**
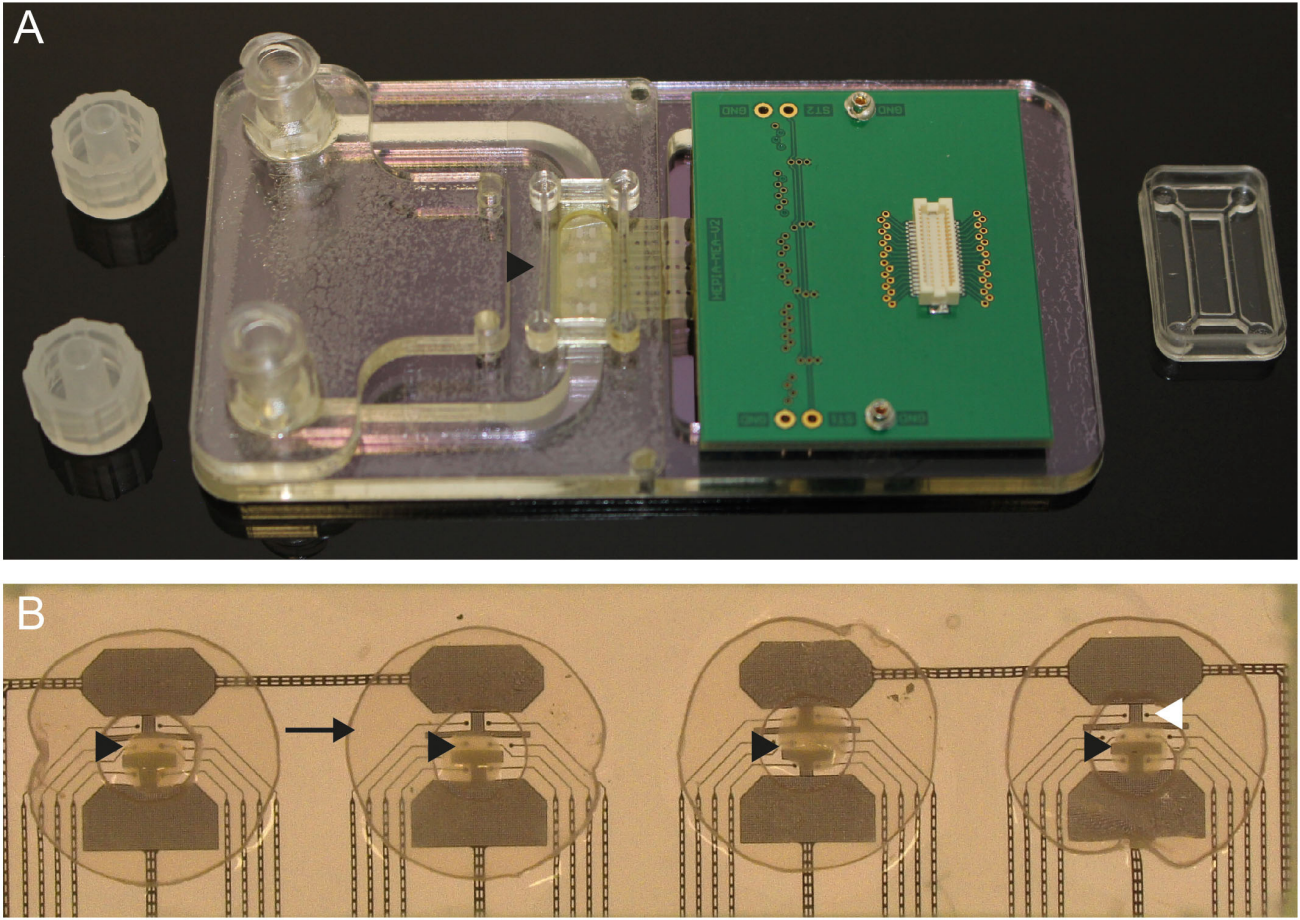
**(A)** Photo of the porous MEA where the black arrowhead shows the position of the recording chamber. **(B)** Close-up view of an MEA biochip where each of the four 3D neural tissues (black arrowheads) are placed onto four areas of eight recording electrodes (white arrowhead). The tissues are stabilized by a perforated “confetti” (black arrow).

The 3D neural tissues sitting on a perforated Sterile Hydrophilic PTFE membrane of 2 mm diameter (named as confetti) (PTFE-005, HEPIA Biosciences) were plated onto porous MEA Biochips comprising four independent recording areas (Figure 2). The experiments were performed in triplicates to reach a total of 12 injured tissues analyzed. The selection of the 3D neural tissues was based on the number of active electrodes showing spontaneous activities with spike frequency > 0.1 Hz before the induction of the trauma (at least present on 20–25 electrodes out of the total 32 electrodes). In total, eight electrodes were in direct contact with each 3D neural tissue, and continuous acquisition and analyses of neural activities were performed using the Spike-on-Chips software. Recording of the spontaneous activity was started after 2 or 3 days to allow the activity of the neural networks to be stable. Control recordings were performed during 10-15 min within the incubator before the induction of the trauma. The MEA biochips were then transferred within the TBI-induction platform, and a single ejection of sterile culture medium was applied for each 3D neural tissue. MEA biochips were then put back into the incubator, and post-trauma recording of spontaneous activities was performed immediately and after 1 h, 24 h, and 48 h (10 min per recording). After the inductions of the traumas, we controlled that the tissues were always in the same position on the MEAs. Furthermore, to ensure that any changes in electrophysiological activity were the result of the trauma induction, we conducted “sham” experiments using the same protocol, e.g., the neural tissue samples were removed from the incubator and allowed to rest on the TBI-induction platform for 2 min without inducing trauma. A total of 12 control neural tissues were analyzed.

### Protein multiplex immunoassay materials

Antigens and antibodies were purchased from HyTest Ltd. Antigen GFAP human recombinant (#8G45); monoclonal mouse anti-human glial fibrillary acidic protein (#4G25) clone 83cc (capture antibody) and clone 81cc (detection antibody); antigen FABP human (#8F65), monoclonal mouse anti-human fatty acid-binding protein (#4F29) clone 28cc (capture antibody) and clone 22 (detection antibody); antigen S100BB homodimer and S100A1B heterodimer human (#8S9h); monoclonal mouse anti-human S100 proteins (#4S37) clone 8B10cc (capture antibody) and clone 6G1cc (detection antibody).

Tau biomarker: Human Tau (total) Calibrator [#C018D-2, Meso Scale Discovery (MSD)]; biotin human Tau Antibody (#C21AGT-3, MSD) and SULFO-TAG anti-human Tau antibody (#D21AGT-3, MSD) were employed as capture and detection antibody, respectively.

### Sample preparation for immunoassays

Before the injury, the 3D neural tissues were individually placed inside the wells of a 24-well plate in a 200 μl culture medium. Immediately before the injury and 2 h and 4 h after the injury, the conditioned culture medium was collected and replaced by a fresh one. After collection, the conditioned culture medium was frozen and stored at −20°C until further processing.

### Protein multiplex immunoassay method

Conditions of the immunoassays: The antigen biomarkers, the samples, and the detection antibodies were diluted in 50 mM Tris with 0.1% BSA and 1 mM CaCl_2_. The blocking solution for the singleplex assay contained 1% BSA and 1 mM CaCl_2_ in PBS 1X.

Protein Multiplex assay: The MSD U-PLEX Development Packs protocol was followed. The biotinylated capture antibodies of h-FABP, GFAP, and S100β were diluted 10 times in the same tube (0.286 μg/ml) with the U-PLEX Stop solution. The U-PLEX provided plate was coated with 50 μl of capture antibody mix solution and incubated for 1 h at room temperature (RT) and 700 rpm. Then, the wells were washed with wash buffer (3x 300 μl of PBS 1x with 0.06% Tween-20), and the mixture of antigen biomarkers or the samples or blank solutions (50 μL), respectively, were added to the wells and incubated (1 h, RT, 700 rpm). After the second washing step (3x 300 μL), 50 μl of the detection antibody mixture was added and incubated (1 h, RT, 700 rpm). After the final washing step (6x 300 μL), 150 μl of read buffer B was added to initiate the signal generation and read-out process (MESO QuickPlex SQ 120, MSD).

Singleplex assay: The MSD R-PLEX Antibody Set Single-plex assay protocol was followed. The GOLD 96-well Small Spot Streptavidin plate (Mesoscale, L45SA-1) was coated with 25 μl of Tau biotinylated capture antibody (200 μl diluted in 3.3 ml of PBS 1X) and incubated for 1 h at RT and 700 rpm. Then, the wells were washed with wash buffer (3x 150 μl of PBS 1X with 0.06% Tween-20), 150 μl of blocking solution was added to the wells, and the plate was incubated (1 h, RT, 700 rpm). The Human Tau Calibrator or the samples and blank solutions (50 μl) were added to the wells and incubated (1 h, RT, 700 rpm). After the third washing step (3x 150 μl), 50 μl of the detection antibody was added and incubated (1 h, RT, 700 rpm). After the final washing step (4x 150 μl), 150 μl of read buffer T 2X was added to initiate the signal generation and read-out process (MESO QuickPlex SQ 120, MSD).

Additional products description and methods are provided in the Supplementary material “Multiplex immunoassay materials” and “Multiplex immunoassay methods.”

### Sample preparation for imaging

Before the injury, the 3D neural tissues were individually placed inside the well of a 24-well plate in a 200 μl culture medium. After the injury, the tissues were incubated for 2 h in a fresh culture medium containing Hoechst 33342 (#H3570, Invitrogen) and Propidium Iodide (PI) (#P5066, Invitrogen). After the staining, 3D neural tissues are fixed in 4% paraformaldehyde for 30 min at RT then washed in phosphate-buffered saline. For optimal imaging, all the samples are cleared by incubation in RapiClear 1.47 (SunJin Lab) for 2 days at RT. The samples were imaged directly within fresh clearing solution by confocal microscopy or embedded in 1% agarose aqueous solution within fluorinated ethylene propylene tubes for OPT. For the latter, the agarose was solidified on ice to limit the rehydration of the tissue and the reversibility of the clearing process. Intact 3D neural tissue was used as negative control and dead 3D neural tissue, e.g., chemically killed by immersion in 4% paraformaldehyde for 12 h was used as positive control. All control samples were processed identically as described previously.

### Confocal imaging

All confocal images of 3D neural tissue were acquired with a TCS SPE microscope (Leica) equipped with an ACS APO 10X 0.3 NA dry objective using Leica LAS × software (Leica). A 405-nm laser was used to excite Hoechst 33342 staining agent (all cell's nuclei), while a 532-nm laser was used to excite PI staining (dead cell nucleus). Z stacks were acquired with a voxel depth of 2.4 μm and an average of 40 sections. In these conditions, the acquisition time was approximately 650 s. All the images are processed using Fiji software.

### 3D projection tomography and analysis

3D imaging was performed using OPT. The setup presented by Schmidt et al. (2021) was adapted to distinguish dead from healthy cells inside the 3D neural tissue. The optical lens was replaced with a commercial telecentric lens (#63-738, Edmund) providing 8 μm spatial resolution. Hoechst staining of all nuclei was excited at 415 nm and acquired between 480 nm and 520 nm using a bandpass filter while PI staining of dead cells' nuclei was excited at 530 nm and acquired beyond 600 nm. The samples were imaged in the water acting as a refractive index matcher using a CMOS camera (#Chameleon3, Flir). Acquisition times for each sample were shorter than 180 s per spectral channel. Supplementary corrections have been implemented in the reconstruction algorithm to take residual mechanical instabilities (Lu and Mackie, 2002) and refractive index artifacts (Liu et al., 2022) into account.

3D visualization and data analysis were performed using the open-source 3D ImageJ Suite software (Ollion et al., 2013). The volumes of the 3D neural tissues were calculated by applying a threshold at 2% of the maximum pixel value and counting the number of voxels above it. The mean diameter of each organoid was deduced from volume assuming spherical geometries. The ratios of dead cells' nuclei into the tissues were calculated for each 3D neural tissue by isolating and counting the cells' nuclei from the two 3D images of the Hoechst and PI staining. We first applied a 3D Gaussian blur filter with a two-voxel kernel to denoise the signal and then used the 3D Maxima Finder plugin with a radius parameter of three voxels to identify the nucleus coordinates. The colocalization of peaks between the two channels was estimated using the 3D Distance Closest plugin with an exclusion distance parameter. This exclusion distance was adapted manually for each acquisition, and the uncertainty on the number of dead neurons was calculated by applying an (-5, +5) interval on this parameter.

### Statistical analysis

Data are presented as means and standard deviations unless indicated otherwise. Statistical differences comparing means were analyzed using a two-tailed Student's *t*-test or ANOVA. Tukey's multiple comparisons test for ANOVA was used to determine the difference between groups and is indicated by an asterisk and black bar. All statistics were performed using GraphPad Prism 9.

## Results

### TBI-induction platform validation

We have developed an integrated platform for inducing traumas on *in vitro* human neural tissues (Figure 1 and Supplementary Figure 1). This platform has several main functionalities, including the precise positioning of samples (via X, Y adjustments) under the microvalve outlet (via Z adjustment), the control of parameters for liquid ejection to induce traumas, the visualization of tissues before, during, and after impact, and the maintenance of sterility for both the microvalve and tissues during injury.

Achieving a controlled induction of mTBI in 3D neural tissue specimens ranging from 0.5 to 1 mm requires precise dosing of force. To determine the optimal parameters for the ejection of culture medium onto 3D neural tissues, we performed a series of tests at varying pressures and distances between the sample and the microvalve nozzle (data not shown). The samples were then incubated in a fresh culture medium containing cell-permeant nuclear staining and cell-non-permeant nuclear staining for 1 h before chemical fixation for live/dead cell analysis. Based on preliminary results, we found that reproducible results were obtained when the samples were positioned 10 mm from the microvalve nozzle, with an open time of 1 ms for the ejection of the culture medium.

We found that the combination of a box enclosure around the TBI-induction platform and a protocol for sterilizing the pneumatic and liquid ejection system was effective in preventing contamination of the injured tissues and enabling analysis during the post-trauma recovery period. Moreover, due to a continuously supplied stock of culture medium in a 5 ml sterile syringe, numerous neural tissues could be injured in succession.

### Electrophysiology characterization of the injured tissue

**Figure 3:**
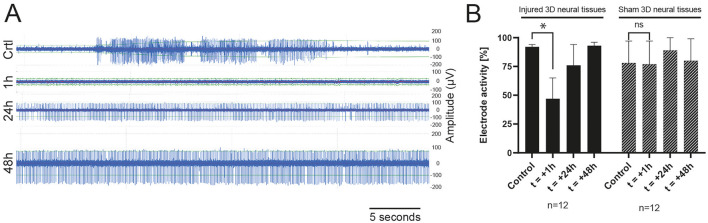
**(A)** Example of raw data during a TBI experiment of spontaneous activity recorded from one electrode 15 min before the induction of the trauma (Control), 1 h, 24 h, and 48 h (top-down) after impact showing a progressive recovery of the electrophysiological activity. **(B)** Histogram of the total active electrode in sham (*n* = 3) or TBI (*n* = 3) experiments in the control condition (before the induction of the TBI), 1 h, 24 h, and 48 h after the trauma.

Figure 3A shows an example of action potentials recorded from the same electrodes over a period of 30 s: before (control), 1 h, 24 h, and 48 h after the induction of the trauma. The control tissue is characterized by a spontaneous activity with amplitudes peak to peak around 200–250 μV. Just after the induction of the injury, we observed a clear abolition of the electrophysiological spontaneous activities, followed by a progressive increase after 24 h, reaching nearly full recovery levels after 48 h. To assess the electrophysiological activity, we combined the number of MEA electrodes that exhibited recorded signals with the number of electrodes that displayed no detectable activity. This approach enabled the determination of the overall activity of the electrodes and the identification of potential alterations in electrophysiological activity (Figure 3B and Supplementary Figure 2). A decrease in activity 1 h after the induction of TBI was observed. Partial recovery was observed after 24 h, with a complete return to control levels obtained at 48 h. The right panel of Figure 3B shows the number of active electrodes recorded in the “sham” experiments. We did not measure any significant decrease in electrode activity under this condition.

### mTBI-related biomarkers released by injured tissue

**Figure 4:**
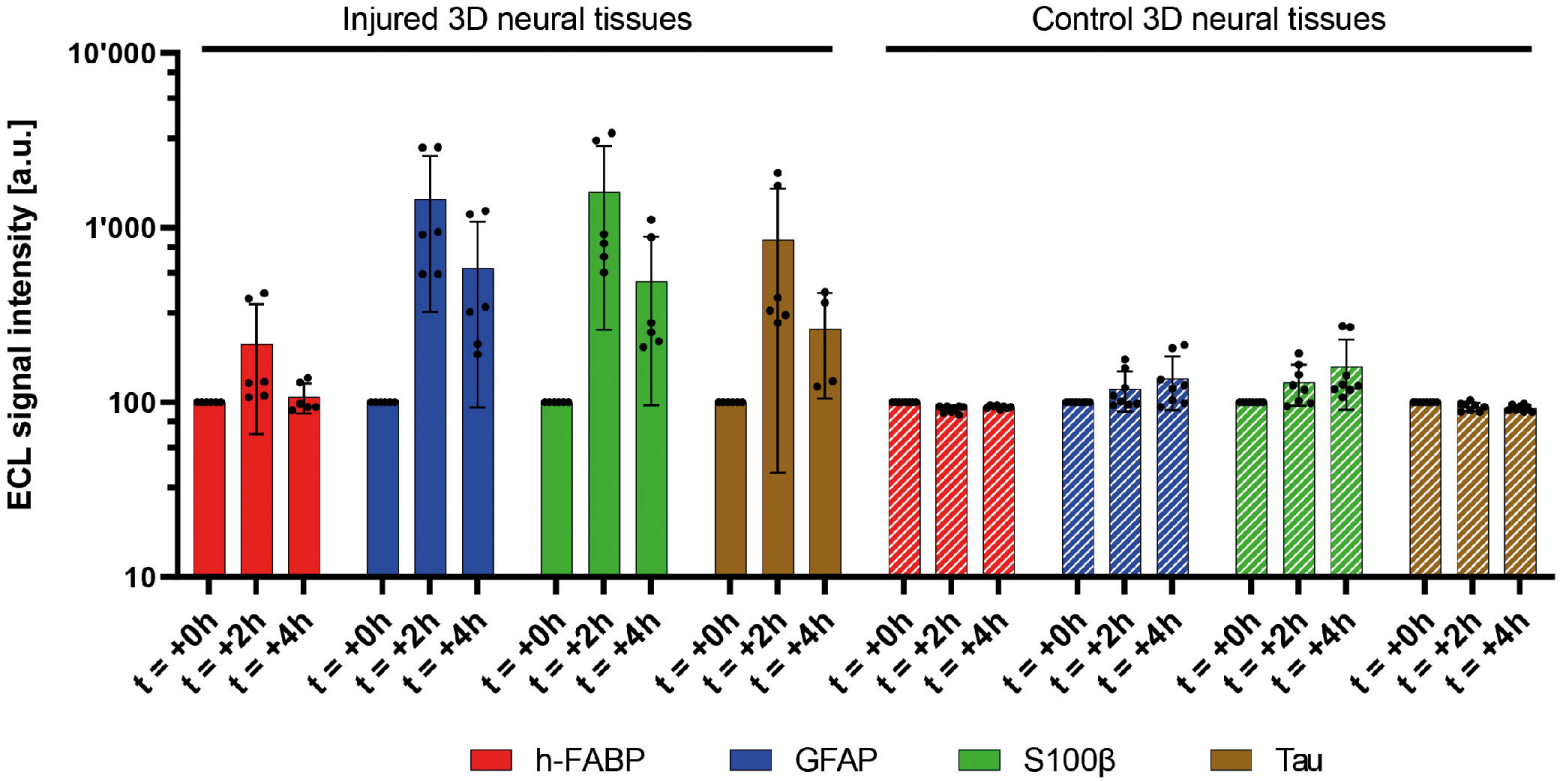
Measured relative signal amplitudes of four mTBI-related biomarkers (h-FABP, GFAP, S100β, and Tau) in injured (five bars, 1,000 μs) and control 3D neural tissues at two discrete **(left)**. Average of duplicates of three independent 3D neural tissues. Mean value (*n* = 8 for control 3D neural tissues, *n* = 6 for injured 3D neural tissues, and *n* = 4 for injured 3D neural tissues at 4h Tau biomarker) ± SD error bars. A significant increase in the release of all biomarkers is observed during the first 2 h (t = +2h) after the pulse (t = 0h). The control neural tissues did not present such biomarker concentration increases in the medium **(right)**.

In this study, multiplex and singleplex electrochemiluminescence immunoassays (ECLIA) were utilized to examine the release kinetics of four protein biomarkers, namely, h-FABP, GFAP, S100β, and Tau, associated with mild traumatic brain injury. Our findings, shown in Figure 4, revealed that the four protein biomarkers were detected in the culture medium after mTBI induction and demonstrated a statistically significant increase compared to the control samples that did not experience any injury or samples before the injury. Specifically, GFAP, Tau, and S100β exhibited a remarkable 15-fold increase at 2 h post-injury, while h-FABP showed a moderate 2-fold increase. Furthermore, 4 h after the injury, there was a sustained release of these three biomarkers into the culture medium, with levels eight times higher than before the injury. Notably, h-FABP concentrations returned to the original background level 4 h later. Additionally, it is worth mentioning that even in the control samples, there was a constant, yet significantly lower release of the four biomarkers into the culture medium. It is also important to mention that even in the control samples, there was a consistent, but significantly lower, release of the four biomarkers into the culture medium. This finding is not surprising since these circulating biomarkers can be detected at sub-pathological concentrations under normal human physiological conditions as well.

### Characterization of the injured neural tissue by optical microscopy

**Figure 5:**
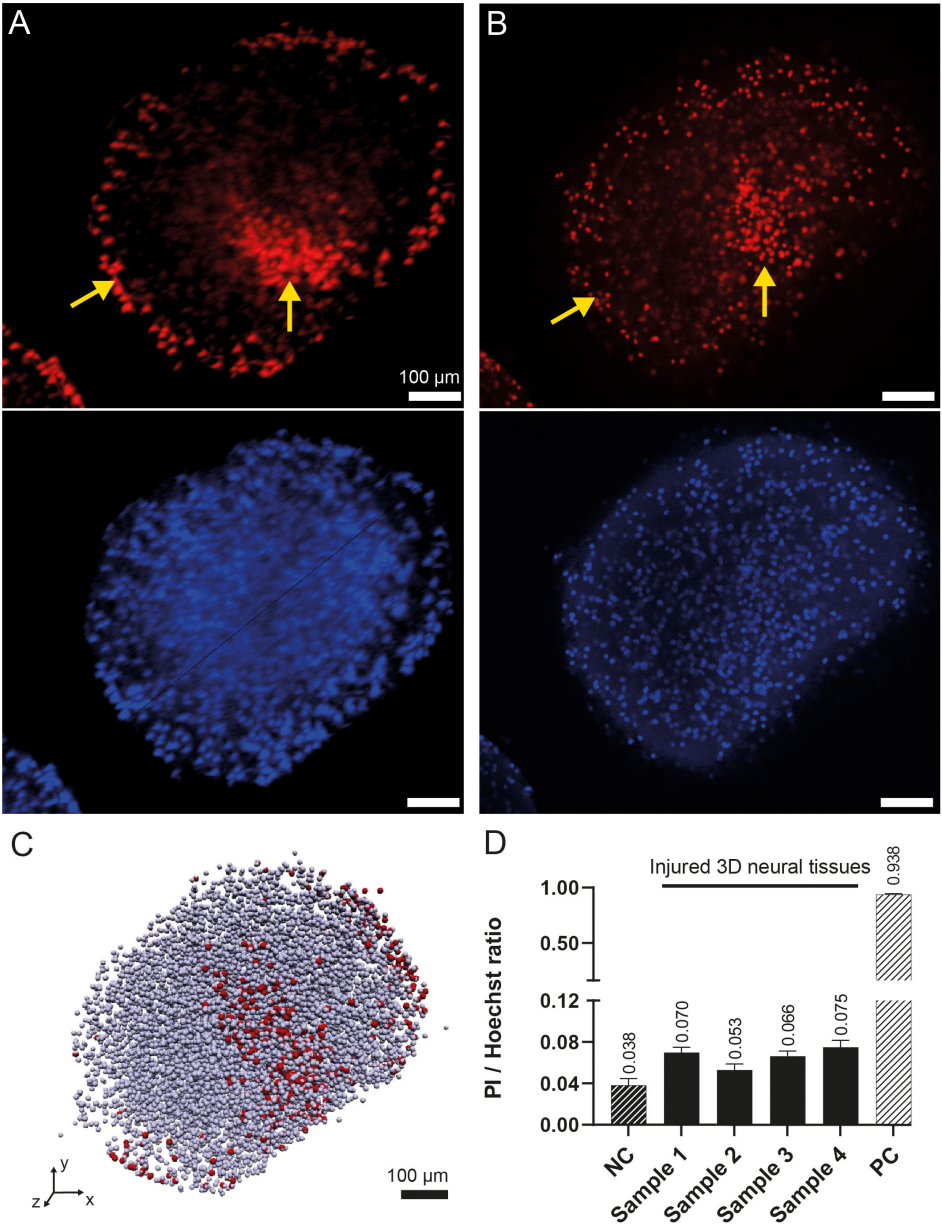
3D rendering of the human 3D neural tissues after TBI impacts. **(A)** Maximum intensity projection of the PI (top) and Hoechst (bottom) fluorescence signals acquired by OPT after integrating the signal over a 100-μm-thick region. The color intensities are saturated to increase the ease of readability. **(B)** Similar view as in **(A)** acquired by confocal microscopy. The yellow arrows show the regions with higher densities of dead cells at the periphery of the tissue and around the main damaged area. **(C)** OPT 3D vectorized model representation of the identified cells. Dead cells are in red, whereas all other cells are in gray. **(D)** OPT computed PI/Hoechst ratios for four injured 3D neural tissue samples. A negative control (NC) organoid was imaged without TBI. A positive control (PC) was imaged after chemical fixation of PI.

Figure 5 illustrates the comparison of 3D neural tissue imaged by OPT and confocal microscopy after a clarity step as described in the material sections. The sample was stained and chemically fixed 2 h after the injury as described in the methods section. All cell nuclei were stained using Hoechst staining (displayed in blue, Figures 5A, B second row), while dead cells were stained using PI staining (displayed in red, Figures 5A, B first row). Confocal microscopy was used as a gold standard to compare the signal of the OPT instrument. However, due to the penetration depth limitation of this imaging method (Pawley, 2006), we were only able to resolve virtual sections of tissue thinner than 200 μm. OPT provided a high spatial resolution of 8 μm and a field of view that enabled whole-tissue imaging. As a result, OPT allowed the identification of individual nuclei, as illustrated on an interactive model (Figure 5C), to calculate the ratio of dead cells, and to determine various structural parameters of both control and injured 3D neural tissues (see Table 1). Specifically, we estimated the mean volume and diameter of the 3D tissues to be 0.15 ± 0.06 mm^3^ and 0.7 ± 0.1 mm, respectively. Additionally, the average nucleus density in the 3D neural tissues was calculated to be 58 kcells/mm^3^, demonstrating the ability of OPT to provide high-resolution imaging and accurate quantification of cellular features in 3D neural tissues.

**Table 1:** 3D neural tissue parameters measured with OPT microscopy.

**Parameters**	**Negative control**	**Positive control**	**Sample 1**	**Sample 2**	**Sample 3**	**Sample 4**	**Mean**	**Std**
Volume (mm^3^)	0.2106	0.1127	0.2324	0.1759	0.1213	0.0726	0.15	0.06
Mean diameter (mm)	0.7382	0.5994	0.7628	0.6951	0.6142	0.5177	0.7	0.1
Number of total nuclei	10495	6107	17271	9625	7636	3734	9k	5k
Number of PI positive nucleus	401	5728	1205	509	507	280		
Nucleus density (nucleus/mm^3^)	49829	54169	74313	54729	62955	51412	58k	9k
Dead cells /total cells ratio	0.038	0.938	0.070	0.053	0.066	0.075	0.07	0.01

Both negative control and positive control were neither considered for ratio means nor for standard deviation (std).

To validate the Hoechst/PI staining method, an analysis of both positive and negative controls was performed. The positive control consisted of 3D neural tissue chemically fixed prior to staining with Hoechst/PI, while the negative control was intact tissue stained before chemical fixation. Analysis showed a live/dead cell ratio of 0.938 ± 0.006 for the positive control, indicating that almost all cells (5,728 of 6,107) were stained with PI. In contrast, the negative control had a much lower ratio of 0.038 ± 0.005. When we imaged the four impacted tissue samples, we observed a significantly higher mean dead cell ratio of 0.07 ± 0.01, compared to the negative control (Figure 5D). These results confirm the effectiveness of the staining method in identifying dead cells in 3D neural tissue.

Qualitatively, the imaging analysis of PI-stained 3D neural tissues revealed the presence of two denser regions, as shown in Figures 5A, B and indicated by yellow arrows. One of these regions was observed in the peripheral cell layer of the tissues, whereas the other was localized deeper within the tissue. While the peripheral signal of cell death was observed in all samples, including control, positive control, and injured tissues, the deeper cell death area was only visible in the injured samples. By isolating the two regions from each other, it was possible to quantify the amount of PI staining in each region. The analysis showed that the mean total amount of signal coming from the main damaged area was nearly equivalent to the amount of signal coming from the tissue border (ratio 1/1.2).

## Discussion

Several *in vitro* and *in vivo* models have been developed to study the cellular and molecular events associated with TBI. Although 2D-based culture models are helpful, they lack the brain's complex 3D cellular organization and extracellular matrix composition. This is a significant weakness as traumatic brain injuries typically involve structural damage to the brain and affect different types of cells and networks of cellular interaction. To overcome this limitation, bioengineered tissues grown in 3D culture systems, mimicking native brain anatomy and physiological responses, are emerging as powerful *in vitro* models to investigate the pathophysiology of TBI (Ramirez et al., 2021b; Silvosa et al., 2022). This led to the development of a new *in vitro* model of TBI combining human cells organized in a 3D neural tissue subjecting to fluid percussion injury. During preliminary experiments, we calibrated the different parameters controlling the microvalve to perform a mild injury on the tissue. These parameters were kept constant for the entire study, and the tissue's response was analyzed with the different read-out modalities.

The occurrence of spontaneous action potentials in mature 3D neural tissues was reported previously (Quadrato et al., 2017), providing confidence in the utilization of electrophysiological recordings for tracking the functionality of neural networks following exposure to trauma. Although there was some variability observed among the various injured tissues, a distinct reduction in spontaneous activity recorded from most electrodes on the MEAs was noted. These observations are in accordance with previous studies carried out *in vivo* (Ding et al., 2011; Johnstone et al., 2013) and *in vitro* (Silvosa et al., 2022). Gradual restoration of spontaneous activity was also observed over time. Other authors reported a loss of activity followed by a slow activity decay to a stable, level plateau approximately 30–40% below reference when working with embryonic cortical tissue from mice (Rogers and Gross, 2019). For this work, we used MEA biochips made of four areas incorporating eight electrodes each. Unfortunately, this MEA design was not fully adapted to the size of the 3D neural tissues. Therefore, different types of MEAs incorporating 32 recording electrodes adapted for a single tissue have been designed to get a more precise mapping of the entire neural network activity of the tissue. Those MEAs will provide the possibility to analyze the neural activity of the directly impacted region compared to activities from more distant areas. Beyond the current study, further electrophysiology experiments and data analysis using these improved MEA biochips are needed to characterize in more detail the effect of impacts on the whole neural network activities (frequency, amplitude, bursts, etc.) and provide valuable information regarding the functional impairment of neural tissue by TBI.

Tissue response to TBI is a mixture of molecular and cellular events. Biomarkers that can track these lesions and inflammatory processes are being explored for their potential to provide objective measures in the evaluation of the injury (Gutierre et al., 2021). In this study, as a preliminary mTBI biomarker panel and based on the cell's distribution in the 3D neuronal tissues, we aimed to detect four proteins known for their clinically relevant relation with mTBI as previously described (Posti et al., 2019; Gutierre et al., 2021). Glial fibrillary acid protein (GFAP) is a structural astrocyte protein while S100β is a calcium-binding protein expressed in cardiac muscles and astroglia among others. Axonal phosphoprotein Tau is expressed in axons and organs like kidneys and liver and, finally, heart fatty-acid-binding protein (h-FABP) is expressed in the heart but also in the brain. To confirm the validity of the presented *in vitro* TBI model, we conducted an analysis of the four protein biomarkers in the culture medium of 3D neural tissue both before and after injury. These biomarkers are not expected to be detectable at a high concentration, which was indeed confirmed in control experiments (Figure 4). Following a TBI impact, the concentration levels of the biomarkers increased and with a certain delay decreased again. So, at least qualitatively a similar kinetic observation is made compared to human *in vivo* TBI (Lagerstedt et al., 2018; Posti et al., 2019; Krausz et al., 2021). In the absence of a blood–brain barrier and vascular circulation (which would dilute/delay the release of biomarkers), a rapid increase in biomarker concentration is expected. A decrease in the concentration relatively soon after the injury, as shown in this study, suggests a minor (or moderate) as opposed to a major injury. However, this would have to be confirmed with more measurement time points (to generate a concentration profile) and an extended observation period to characterize for instance when post-injury also GFAP, S100β, and Tau return to baseline concentrations. In fact, one would expect the slope of increase and decrease as well as the area under the curve to be different as a function of impact magnitude on the tissue. Repetitive vs. single trauma may also show different release kinetics. It is important to note that the high sensitivity of the ECLIA developed allows us to measure more subtle concentration changes, thus monitoring less significant injuries. It is also noteworthy that the injured 3D neural tissues seemed to release lower quantities of neuronal biomarkers, such as h-FABP, compared to glial ones (GFAP and S100β) and axonal (Tau). Further investigations are necessary to improve the quantification of these biomarkers and to find out whether these observations are indeed due to the vulnerability of certain neuronal structures, the relative number of corresponding cell types, or a combination of both. The slight and steady rise of biomarkers in the controls may be correlated with OPT results obtained. It is indeed observed that, even in the absence of injury, a small fraction of the cells is dead at the periphery of the 3D neural tissues that could lead to the release of biomarkers and therefore increase the level of our studied proteins in the culture medium. Some experiments are ongoing to evaluate this effect.

Finally, to characterize the injured tissue, a mesoscopic imaging method, OPT, that was recently developed (Schmidt et al., 2021) to image millimetric tissue with a micrometric resolution, was used. With the 3D images acquired by OPT, it was possible to determine a mean 3D neural tissue diameter of 0.7 ± 0.1 mm and a mean cell density of 58 kcells/mm^3^. This latter parameter is in good accordance with values found for the mouse brain organoid (Keller et al., 2018). In addition, the measured ratio of damaged cells 2 h after the impact (*t* = +2h) is significantly higher than what was measured without injury (negative control). As PI staining is membrane impermeant and should therefore be excluded from viable cells, we can assume that the cells detected as dead by OPT will not recover with time. Therefore, the extra dead cells ratio measured in the injured 3D neural tissues with respect to the negative control is attributed to the induced trauma. The spatial distribution of injured cells throughout the 3D neural tissue also allowed us to identify a main damaged area that is confined within the tissue or to the periphery, but within a limited solid angle. The observation of a permanent reduction of healthy cells after a head injury is in some ways at odds with the other results, which all show almost complete signal recovery over time. We explain this behavior by the fact that OPT measures the state of the entire sample at a specific time, whereas electrophysiological characterization only reads the signal from cells in contact or close to the electrodes and immunoassays measure protein diffusion over time and through the entire tissue. A better understanding of the diffusion process, in this case, requires more research and simulations.

## Conclusion

The *in vitro* TBI model we have developed in this study allows not only to reproducibly induce injuries on human 3D neural tissues but also a comprehensive characterization of injured tissue during the acute and recovery phases. Due to the versatility of the developed platform, it was possible to show a clear abolition of electrophysiological spontaneous activities just after the induction of the injury, followed by a progressive increase in activity after 24 h and full recovery after 48 h. Additionally, four protein biomarkers associated with mild traumatic brain injury exhibited a statistically significant increase in their release into the culture medium compared to the control samples. Finally, the optical projection tomography provided images of injured areas with cellular resolution and allowed an accurate quantification of cellular features in 3D neural tissues.

In the next step, we plan to expand the range of data points after TBI induction to cover immediate and long-term responses, including data points that capture the first few minutes post-trauma and responses that occur over weeks or months. Finally, it is planned to further improve the system by the development of a more sophisticated biological model by the addition of different types of membrane-like structures on the surface of the brain parenchyma, to mimic the presence of a skull or meninges, which at least partially protects the brain from external forces. Therefore, the impacts of ejected culture medium will be more diffuse/distributed (as opposed to focusing on a small area) and would thus recreate more realistically concussion events such as the ones occurring in *in vivo* experiments.

## Data availability statement

The raw data supporting the conclusions of this article will be made available by the authors, without undue reservation.

## Author contributions

LS, AR, and MP designed the study. CL-F, YN, OR, DP, and CS collated the data, carried out data analyses, and produced the initial draft of the manuscript. MH, LG, MJ, JE, LS, AR, and MP helped perform the analysis with constructive discussions. All authors have read and approved the final submitted manuscript.

## Multiplex immunoassay materials

All chemicals were used as received without further purification and all aqueous solutions were prepared with MQ water. U-PLEX Development Pack, 6-Assay (#K15231N-2), GOLD 96-well Small Spot Streptavidin Plate (#L45SA-1), GOLD SULFO-TAG NHS-Ester lyophilized (#R91AO-1), Gold read buffer B (#R60AM-2), read buffer T 4X (#R92TC), U-PLEX Stop Solution (#R50AO-1) were purchased from MSD. Other materials include Pierce Antibody Biotinylation Kit for IP (#90407, Thermo Scientific, Waltham), bovine serum albumin fraction V (#10735086001, Roche Diagnostics), PBS 10x pH 7.4 phosphate saline buffer (#70011-036, Gibco), Tween-20 [#P1379-100 mL, Merck (Sigma-Aldrich)], CaCl_2_ x 2H_2_O (#223506, Fluka), Trizma base (#1002134476, Sigma-Aldrich,), Zeba Spin desalting columns 7K MWCO, 0.5 mL (#89882, Thermo Fisher).

## Protein multiplex immunoassay method

Capture antibody biotinylation and linker-coupled antibody solutions: The capture antibodies (h-FABP, GFAP, and S100β) were biotinylated with the EZ-Linker NHS-PEG4-Biotin antibody Biotinylation Kit from the Pierce™. The concentration of the antibody solutions was 1 mg/mL, and 3.14 μL of NHS-PEG4-Biotin solution was added to the diluted antibodies. The final concentrations of the biotinylated capture antibodies were measured at OD280 using the Nanodrop OneC Microvolume UV-VIS Spectrophotometer (Thermo Fisher Scientific). Each biotinylated capture antibody was coupled with a determined linker from MSD to obtain a final antibody-linker concentration of 2.86 μg/mL following the MSD protocol (MSD U-PLEX Development Pack). Tau capture antibody was diluted (200 μL in 3.3 mL of PBS 1X) and used without further purification (MSD R-PLEX Antibody Sets Singleplex Assays).

Detection antibody labeling: Detection antibodies of all three biomarkers were conjugated with the ${\text{Ru(bpy)}}_{3}^{2+}$-label using the GOLD SULFO-TAG NHS-Ester reagent provided by MSD (#MSD GOLD SULFO-TAG Conjugation Quick Guide). A challenge ratio of 50:1 was used for all detection antibodies (h-FABP, GFAP, and S100β), while the labeling incorporation ratio was calculated by measuring the OD_455_ values using the NanoDrop OneC Microvolume UV-VIS Spectrophotometer (Thermo Fisher Scientific). The calculated label ratio for h-FABP, GFAP, and S100β detection antibodies were 14:1, 13:1, and 19:1, respectively. The Tau detection antibody was used 100-fold diluted without further modification or purification.

## Supplementary material

Supplementary Figure 1 Picture showing the microvalve (white arrow) attached to an XYZ translation stage mounted on an inverted microscope. The 3D neural tissues are disposed in a 24-well plate to perform the injury.

Supplementary Figure 2 Example of raster plots of 4 groups of eight electrodes. Each group of 8 electrodes, displayed in a different color (red, green, blue, and black), recorded one 3D neural tissue. The data were acquired during a TBI experiment. On the first row, one can observe the spontaneous and synchronous activity 15 min before the trauma induction (Control). After the trauma, e.g., 1 h, 24 h, and 48 h **(top-down)** after impact, one can observe a progressive recovery of the electrophysiological activity.
