# Supplementary figures and images for: Opening the black box of traumatic brain injury: a holistic approach combining human 3D neural tissue and an in vitro traumatic brain injury induction device
Source: Front Neurosci. 2023 Jun 15;17:1189615. doi: 10.3389/fnins.2023.1189615 (PMC10308006; doi:10.3389/fnins.2023.1189615)

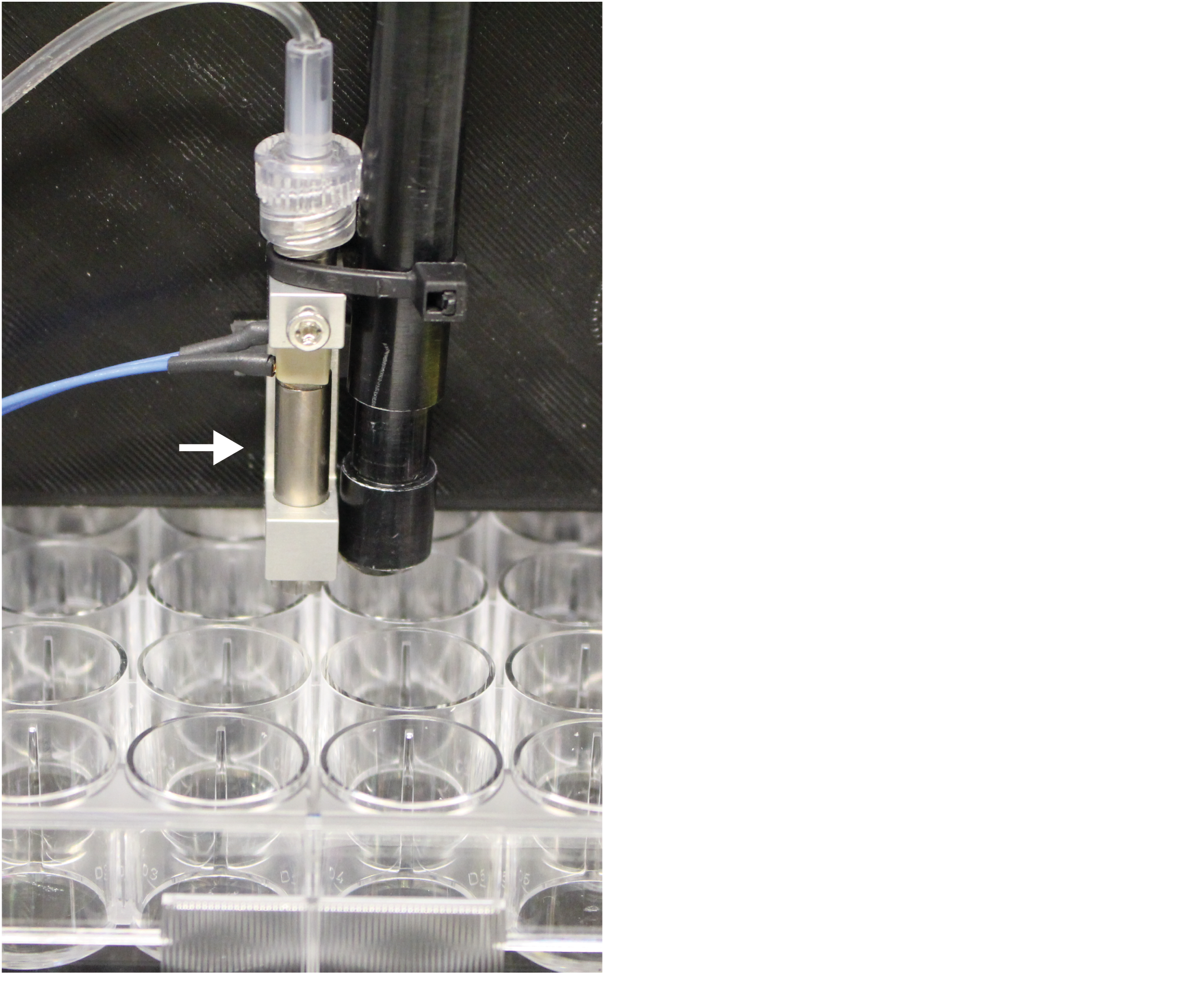

Supplement: Supplementary Figure 1 — Picture showing the microvalve (white arrow) attached to an XYZ translation stage mounted on an inverted microscope. The 3D neural tissues are disposed in a 24-well plate to perform the injury. [file Image_1.TIF]

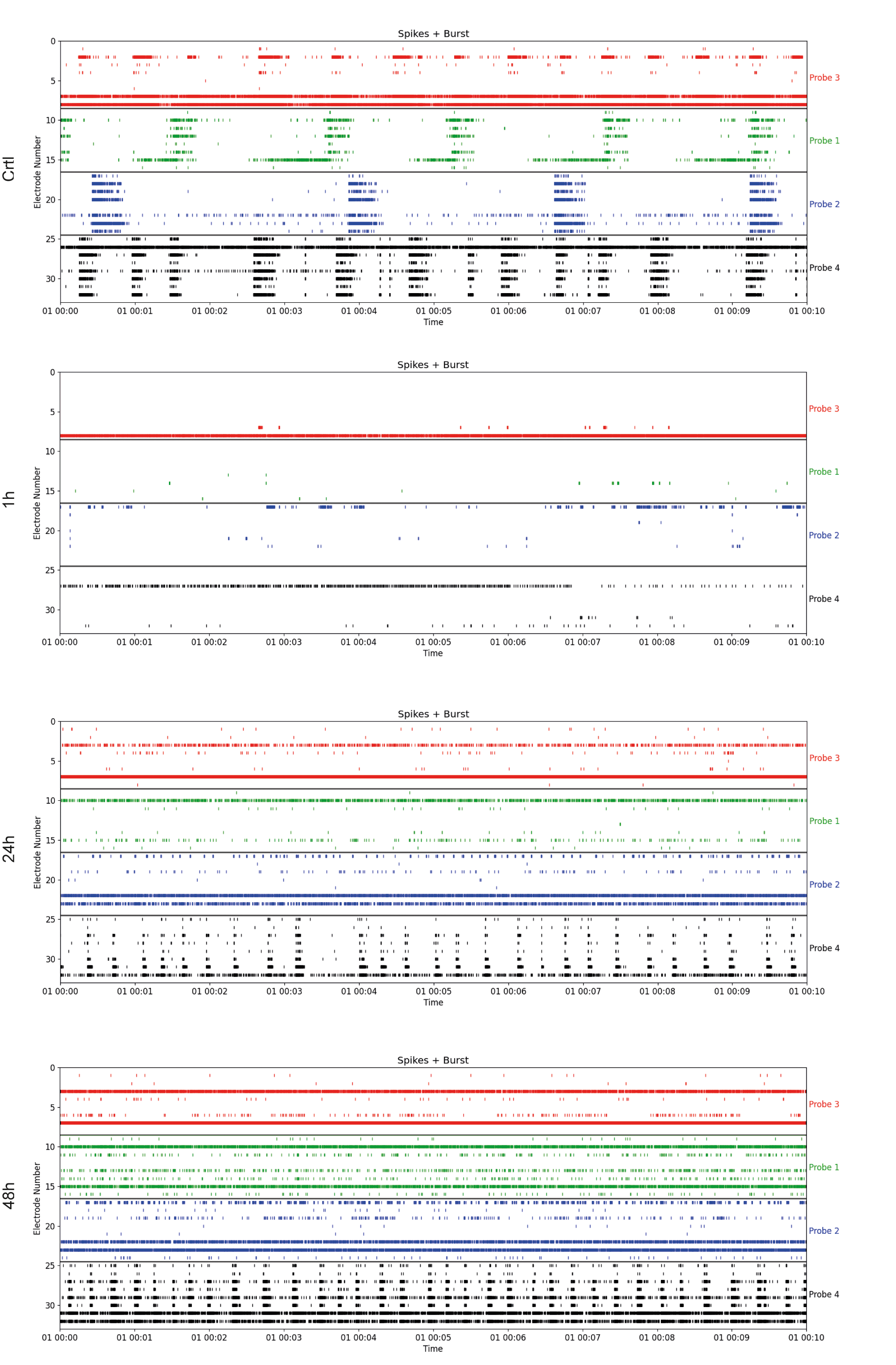

Supplement: Supplementary Figure 2 — Example of raster plots of 4 groups of eight electrodes. Each group of 8 electrodes, displayed in a different color (red, green, blue, and black), recorded one 3D neural tissue. The data were acquired during a TBI experiment. On the first row, one can observe the spontaneous and synchronous activity 15 min before the trauma induction (Control). After the trauma, e.g., 1 h, 24 h, and 48 h (top-down) after impact, one can observe a progressive recovery of the electrophysiological activity. [file Image_2.TIF]
